# Microwave Irradiation Process for Al–Sc Alloy Production

**DOI:** 10.1038/s41598-020-59664-2

**Published:** 2020-02-14

**Authors:** Satoshi Fujii, Eiichi Suzuki, Naomi Inazu, Shuntaro Tsubaki, Jun Fukushima, Hirotsugu Takizawa, Yuji Wada

**Affiliations:** 1 Department of Applied Chemistry, Graduate School of Science and Engineering, Tokyo Institute of Technology, 2-12-1 Ookyama, Meguro-ku, Tokyo, 152-8550 Japan; 2 Department of Information and Communication System Engineering, National Institute of Technology, Okinawa College, 980 Henoko, Nago-shi, Okinawa, 905-2192 Japan; 3 Department of Applied Chemistry, Graduate School of Engineering, Tohoku University, 6-6-07 Aoba, Aramaki, Sendai, Miyagi 980-8579 Japan

**Keywords:** Microwave chemistry, Chemical engineering

## Abstract

Scandium is being explored as an alloying element for aluminium alloys, which are gaining importance as high-performance lightweight structural alloys in the transportation industry. Sc-rich ScAlN thin films show strong piezoelectricity and can be fabricated on a hard substrate for use as wideband surface acoustic wave filters in next-generation wireless mobile communication systems. However, the use of ScAlN thin films in microelectromechanical system devices is limited by the high cost of metallic Sc, which is due to the difficulty in smelting of this material. Here, we propose a novel microwave irradiation process for producing Al-Sc alloys, with Mg ions as a reducing agent. Although scandium oxide is thermodynamically stable, intermetallic Al_3_Sc is obtained in high yield (69.8%) via a low-temperature (660 °C) reduction reaction under microwave irradiation. Optical spectroscopy results and thermodynamic considerations suggest a non-thermal equilibrium reaction with the univalent magnesium ions excited by microwave irradiation.

## Introduction

Scandium is the 31^st^ most abundant element in the earth’s crust, with a Clarke number of 22 ppm^1^. Sc metal was successfully extracted by Fisher *et al*. in 1937, using an electric field melting system, and high-purity Sc (99%) was produced in 1965^2^. However, pure Sc has become available only recently, and it is very expensive. Industrial research into Sc commenced with a patent from Alcoa, involving the addition of Sc to an Al alloy^3^. Compared to other rare earth metals, Sc dramatically improves the metal material properties when added to Al alloys;^4^ the addition of Sc greatly affects the recrystallization of pure Al and Al alloys^5^. It is also known that Sc strengthens the Al–Mg alloy to a greater extent than Zn, Cr, and Mn^6^. Further, the addition of a small amount of Sc to the commercial 7010 alloy reduces its susceptibility to hot cracking during the solidification of welds^7^. Because of these advantages, Al–Sc alloys are being produced commercially for use in fabricating expensive products, such as aviation and aerospace components, bicycle frames, and baseball bats^8,9^. However, the high cost associated with the smelting of Sc necessitates the need for an alternative inexpensive process, to extend the application of Sc as an energy-saving structural material.

Recently, Sc-rich ScAlN thin films have attracted considerable research attention because of their strong piezoelectricity. Akiyama *et al*. found that the piezoelectricity of Sc_x_Al_1-x_N thin films increased monotonically with an increase in the Sc concentration, *x*^10,11^, reaching the maximum value at *x* = 43 at.%, where the piezoelectric coefficient, *d*_33_, was five times that of pure AlN^12,13^. Hashimoto *et al*. reported that a surface acoustic wave (SAW) resonator based on the ScAlN/6H-SiC structure exhibited resonance *Q*_*r*_, antiresonance *Q*_*a*_, and *K*^2^ values of 340, 240, and 4.5%, respectively, at 3.8 GHz. A SAW based on the ScAlN/diamond structure exhibited *Q*_*r*_ and *K*^2^ values of 520 and 6.1%, respectively, at 3.7 GHz^12,13^. These values suggest that Sc_x_Al_1-x_N thin films formed on a hard substrate are suitable candidates for wideband SAW filters in next-generation wireless communication systems. The potential applications of Sc, e.g., ScAlN thin films for acoustic microelectromechanical system **(**MEMS) devices and lightweight Al–Sc alloys, are restricted by the high-cost, difficult sintering process of metallic Sc.

Thermal reduction is the most common method for producing Sc. Scandium oxide, the raw material for this process, is thermodynamically stable. Hence, it is converted into scandium fluoride, which is easily reduced at 1873 K using metallic calcium as the reducing agent, according to the following reactions^14^:

1 $${{\rm{Sc}}}_{2}{{\rm{O}}}_{3}+6{\rm{HF}}- > 2{{\rm{ScF}}}_{3}+3{{\rm{H}}}_{2}{\rm{O}}$$

2 $$2{{\rm{ScF}}}_{3}+3{\rm{Ca}}- > 3{{\rm{CaF}}}_{2}+2{\rm{Sc}}$$

Because this process involves fluoridation, it is expensive and environmentally unfriendly. Furthermore, some of the calcium remains as an impurity. Hence, various other processes for smelting Sc have been reported^15,16^. For example, Sc can be dissolved in an Al melt by reducing Sc_2_O_3_ directly in the melt^17^. Harata *et al*. demonstrated that Al–Sc alloys can be directly produced via calciothermic reduction, using Ca vapour as the reduction agent and Al as the collector metal^16^. In this method, a mixture of Sc_2_O_3_, Al, and CaCl_2_ in a tantalum crucible with Ca was placed inside a stainless-steel container, and the feed mixture was reacted with Ca vapour at 1273 K for 6 h. The long reaction time and high temperature involved render this method unsuitable for the cost-effective production of Al–Sc alloys. Harata *et al*. also reported the electrochemical production of Al-Sc alloys in CaCl_2_-Sc_2_O_3_ molten salt. Using this method, Al_3_Sc with 6 mass % as a speculated value from the phase diagram was obtained. Due the high temperature of 1173 K employed for melting the salt and the low yield of the Al-Sc alloy, the energy efficiency of this method is not high enough for mass production^18^.

Chemical reactions performed under microwave irradiation often have high rates and high selectivity, allowing the use of a smaller reactor and a more energy-efficient process as compared to conventional heating. Therefore, microwave chemical processing and synthesis have attracted significant attention as a means of improving process efficiency and conserving energy towards ‘green chemistry’ and ‘green engineering’^19–21^. Some researchers have also proposed smelting of the metal using microwave heating to reduce CO_2_ emissions^22,23^. We previously demonstrated the feasibility of microwave irradiation for the smelting of metal, in which we obtained 1.7 g of Mg metal with high yield (71%) and lower energy consumption (one-third of that required for the conventional Pidgeon process)^24^. In addition, we demonstrated that microwave-based carbothermic reduction of ScF_3_ yielded a Sc metal thin film, based on only the X-ray diffraction (XRD) data for the crystalline Sc metallic phase^25^.

In this study, we investigated a new microwave process for producing Al–Sc alloys with lower energy inputs and reduced greenhouse gas emissions as compared to standard methods. The following chemical reaction was carried out using microwave radiation as the heat source:

3 $${{\rm{Sc}}}_{2}{{\rm{O}}}_{3}({\rm{s}})+{\rm{Mg}}({\rm{g}})+{\rm{Al}}({\rm{s}})- > {\rm{Al}} \mbox{-} {\rm{Sc}}\,{\rm{alloy}}\,{\rm{metal}}+{\rm{MgO}}$$

**Figure 1:**
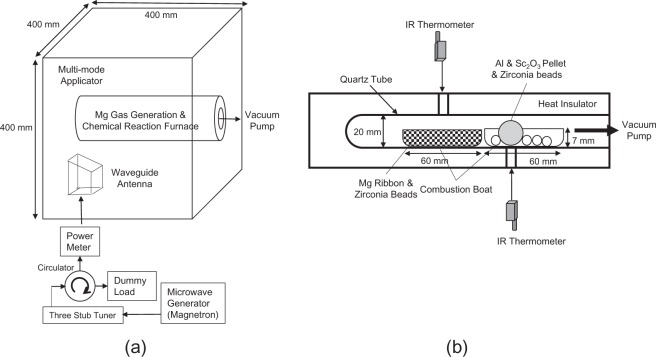
(**a**) Schematic of the experimental setup for our Al–Sc production process, comprising a multi-mode applicator (400 mm^3^), crucible, stirrer fan, and magnetron microwave source. (**b**) The combustion boat for the reduction reaction (containing a briquette of Al and ScO_3_ particles) and the combustion boat in which the Mg vapour is generated (containing Mg ribbon and zirconia beads) are located at the centre of the cavity in a quartz tube under vacuum.

**Figure 2:**
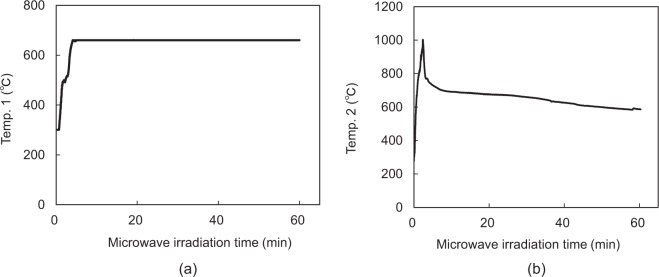
(**a**) Temp.1: Temperature of the crucible containing the Al/ScO_3_ briquette as a function of the microwave irradiation time to maintain the temperature at 660 °C. (**b**) Temp.2: Temperature of the mixture of Mg ribbon and Zr beads as a function of microwave irradiation time.

The experimental setup for our process is shown in Fig. 1. In our experiments, the temperature of the crucible containing the briquette of aluminium and scandium oxide particles was adjusted to the reduction temperature by microwave irradiation. Simultaneously, the temperature of an adjacent crucible containing Mg metal and zirconia balls was increased to the level at which Mg was vaporised. Figure 2 shows the temperature of the crucible for the reduction reaction, as measured using an infrared sensor, and the microwave power as a function of the irradiation time in Eq. (3). Figure 2(a,b) show the temperatures of the crucible used for the reduction reaction and the Mg vapour source as a function of the irradiation time. As seen in Fig. 2(a), the temperature of the reduction reaction in the region of the crucible where the sample and zirconia beads were located was maintained at 660 °C (using a PID to control the microwave incident power). Figure 2(b) reveals that the temperature of the Mg vapour source exceeds the boiling point (650 °C) within 5 min, at the time of Mg vapour generation, and then, the Mg vapour expands to the crucible for the reduction reaction.

**Figure 3:**
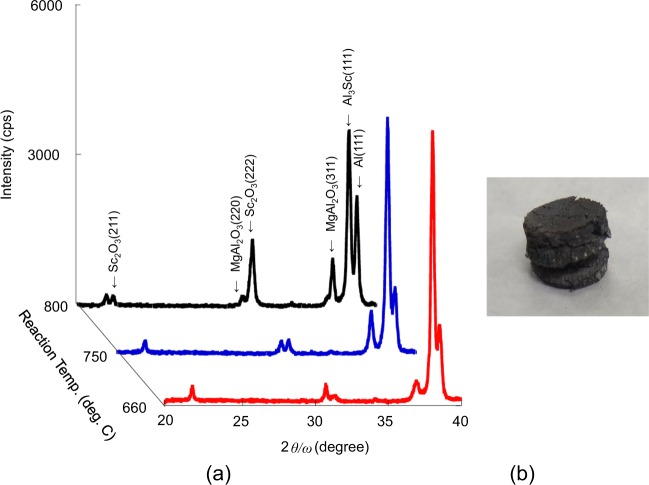
(**a**) XRD patterns of the mixture of residues and products for reaction temperatures of 660, 750, and 800 °C for the chemical reaction shown in Eq. (3). (**b**) Photograph of the briquette after the reduction reaction.

**Figure 4:**
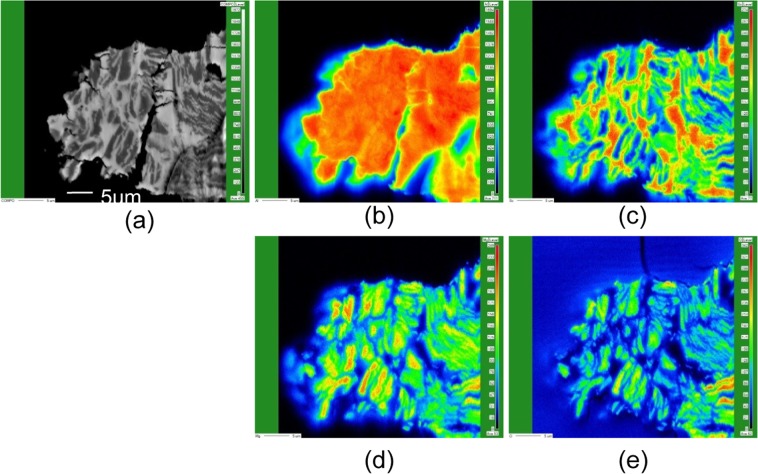
(**a**) SEM image of the reaction product. Intensity distributions of (**b**) Al, (**c**) Sc, (**d**) Mg, and (**e**) O.

Figure 3 shows the XRD results of the products for reaction temperatures of 660, 750, and 800 °C and an image of the briquette after the reduction reaction. For the XRD result of the product with the reaction temperature of 660 °C, a strong peak at *2θ* = 37.93°, attributed to the (111) plane of Al_3_Sc, which was obtained here for the first time by microwave irradiation at 660 °C, was observed in the XRD pattern. The peaks at *2θ = *22.22° and 31.63°, which were assigned to the (211) and (222) planes of scandium oxide, respectively. Simultaneously, the temperature of an adjacent crucible containing Mg metal and zirconia balls was increased to the level at which Mg was vaporised. In addition to unreacted Sc_2_O_3_, only a spinel (MgAl_2_O_4_) compound was identified as the side product. As shown in Fig. 3, the intensity of the XRD peak of the spinel decreases with the reaction temperature. It is possible to eliminate the product of the spinel by lowering the reaction temperature. Because the experimental apparatus used controls the vaporizing of magnesium and the reaction temperature with a single microwave source, the reaction temperature cannot be decreased to 660 °C to vaporize magnesium. Figure 4 shows the electron probe micro analysis (EPMA) results of the cross-sectional area of the reaction product particles of about 50 μm. As shown in this result, Al exists at the location where Sc is present, thereby suggesting that Mg and O exist and confirming the presence of Al_3_Sc. Quantitative analysis was performed using inductively coupled plasma-optical emission spectrometry (ICP-OES) and XRD with internal standard addition for estimating the amount of each product (such as oxides and intermetallic compounds) formed in this reaction. The concentration of each element in the reaction product was confirmed by ICP-OES. The concentrations of the spinel and Sc_2_O_3_ were estimated to be 12.1% and 3.3%, respectively, by the XRD method. The concentrations of all elements and compounds in the product are listed in Table 1. We could not detect the peak due to the presence of MgO because the film thickness was too small to be detected by XRD, and the uncertainty in the measurements increased when the spinel content exceeded 10% when using the internal standard addition method^26^. The results showed a 69.8% yield of Al_3_Sc through this experiment.

**Table 1:** Relative concentrations of the elements and compounds in the reaction product.

	ICP-OES data (wt.%)	Estimated concentration(wt.%)
Element		
Al	60.8	
Sc	21.5	
Mg	6.09	
O		11.0
Compound & Element		
MgAl_2_O_4_*		12.1
Al3Sc		54.3
Al		21.3
Sc_2_O_3_*		3.3
MgO*		6.7
Total		97.6

*The amounts of the spinel and Sc_2_O_3_ were determined by the XRD internal standard addition method using a commercial sample of the spinel and Sc_2_O_3_, respectively, as reference samples. Based on the uncertainty of this estimation method, the total amount of all compounds and elements was under 100%. Due to the low Mg content and low-intensity MgO peak in the XRD measurement, the amounts of MgO and Mg were not determined precisely at this moment.

**Figure 5:**
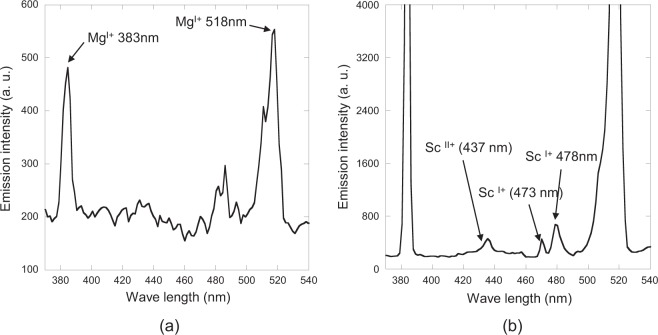
Optical spectroscopy measurement results (**a**) after microwave irradiation of 156 s, (**b**) immediately after microwave irradiation (after 157 s).

The generation of Mg vapour, as explained earlier, in the form of the green plasma was observed visually just after the temperature exceeded 660 °C in the region of the crucible containing the reaction mixture. Optical spectroscopy measurements were carried out on a crucible with the same configuration under microwave irradiation to understand the effect of the plasma on the reaction. The details of this experimental set up will be described later in this section. Figure 5 (a) shows the results of the optical spectroscopy measurements just after the green plasma was generated after 156 s of microwave irradiation. The optical spectrum showed two peaks at 383 nm and 518 nm, which were attributed to emission from univalent magnesium based on the atomic spectra database of NIST^27^. Further, optical spectroscopy measurements were carried out every 0.5 ms after microwave irradiation was started. Figure 5 (b) shows the results of the optical spectroscopy measurement after 157 s after microwave irradiation was started. The peaks at 473 and 478 nm were attributed to univalent scandium. In addition, the peak at 437 nm was attributed to divalent scandium. This result strongly suggests that a non-thermal equilibrium reaction (Eq. (3)) occurred due to the presence of plasma radicals, in which the magnesium atoms were excited by the microwave irradiation.

A thermodynamic study was carried out using the thermodynamic data for scandium oxide and magnesium oxide from the integrated thermodynamic databank system FactSage^28^. The Gibbs free energy change for reaction (4) is 330 kJ/mol at 660 °C, implying that the thermal equilibrium reaction does not occur at this temperature.

4 $${{\rm{Sc}}}_{2}{{\rm{O}}}_{3}+3{\rm{Mg}}- > 2{\rm{Sc}}+3{\rm{MgO}}\,\Delta G=+\,330{\rm{kJ}}/{\rm{mol}}\,{\rm{at}}\,660^\circ {\rm{C}}.$$

Therefore, considering that the plasma emission is due to the Mg ion formation, the ionization energy of Mg is calculated as 8.76 eV and the Gibbs free energy change for reaction (5) as 2536 kJ/mol^29^.

5 $$3{\rm{Mg}}-\, > 3{{\rm{Mg}}}^{+}\,\Delta G=\,+2536{\rm{kJ}}/{\rm{mol}}.$$

After reworking Eq. (4) using the data for (5), we get Eq. (6), for which the Gibbs free energy change is −2200 kJ/mol at 660 °C. This means that reaction (5) occurs when plasma is generated as a result of the ionisation of Mg.

6 $${{\rm{Sc}}}_{2}{{\rm{O}}}_{3}+3{\rm{Mg}}-\, > 2{\rm{Sc}}+3{\rm{MgO}}\,\Delta {\rm{G}}=-\,2200\,{\rm{kJ}}/{\rm{mol}}\,{\rm{at}}\,660^\circ {\rm{C}}.$$

Therefore, the excess energy of −2200 kJ/mol seems to be used for the production of Al_3_Sc and MgAl_2_O_4_. Because the binary Al–Sc phase diagram shows that only a small amount of Sc metal is thermodynamically dissolved at ~660 °C, the eutectic point of Al_3_Sc is 1320 °C^30^, and the melting point of MgAl_2_O_4_ is 2100 °C^31^. Here, a non-thermal equilibrium reaction occurred, and microwaves or plasma radicals in which a magnesium atom and electron were excited by microwaves were considered to compensate for the lack of sufficient reduction reaction energy. To confirm the influence of this magnesium ion or radical, the generation of plasma in the reduction reaction under microwave irradiation was studied by optical spectroscopy.

This work demonstrates a novel process for the production of Al–Sc alloy by microwave irradiation, which is expected to reduce energy consumption and simplify the processing equipment, as the temperature we employed (660 °C) is the lowest among those reported to date. We believe that unreacted scandium oxide can limit the reaction to 100% by optimizing the reaction conditions with lower reaction temperature, and magnesium oxide as a side product can be easily removed with hydrochloric acid. Experimental results confirmed that a non-thermal equilibrium reaction involving scandium oxide occurred because of the generation of plasma radicals and electrons, in which the magnesium atoms were excited by the microwaves.

## Methods

Figure 1 shows the experimental setup consisting of a multi-mode applicator (400 mm^3^), a stirrer fan, a magnetron as a microwave source, a rotary pump, a quartz glass tube, an infrared temperature measurement system (FTK9-R, Japan Sensor Corp.), two alumina combustion boats (located in the quartz glass tube), the briquette (composed of Sc_2_O_3_ and Al particles), and magnesium ribbon (placed in the alumina combustion boats). Aluminium particles (0.588 g; 22 mmol; size: 53–106 μm) and scandium oxide particles (0.300 g; 2.2 mmol; size: 45 μm) were mixed, poured into a stainless-steel die, and subjected to a pressure of 9.81 MPa to obtain a briquette (diameter: 10 mm and height: 5 mm). The briquette was placed in the alumina crucible with the zirconia beads. Zirconia beads were placed in the other alumina crucible within a roll of magnesium ribbons. These combustion boats had a length, width, and height of 60 mm, 10 mm, and 7 mm, respectively. The temperatures of the two combustion boats were measured through a hole in the applicator using an infrared temperature measurement system. For the reaction described by Eq. (3), a microwave power of approximately 300 W was applied to maintain the crucible temperature at 660 °C for 1 h, using a multi-mode applicator. Before applying the microwave power, the quartz glass tube was evacuated to a low pressure of 2 Pa; the vacuum pump was stopped during the reaction.

**Figure 6:**
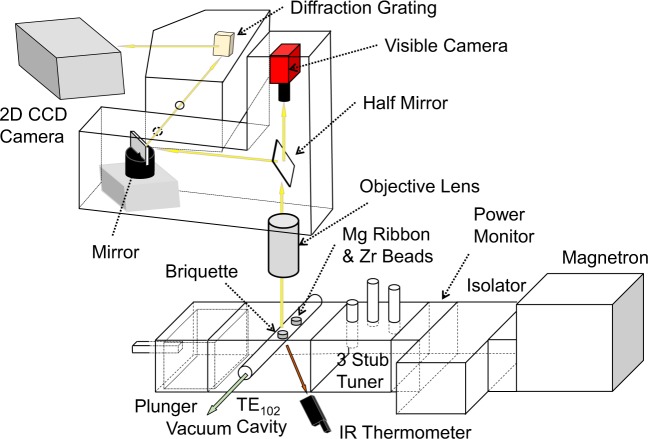
Experimental setup for the optical spectroscopy measurements.

ICP-OES (Agilent technology, ES720) was carried out to determine the elemental concentration. Figure 6 shows the experimental setup for the optical spectroscopy measurements, which includes a microwave generator with a phase-locked loop (PLL) oscillator, a power amplifier module (FSU-201VP-02, Fujidenpa Corp.), a plunger for impedance matching, a waveguide cavity with the TE102 mode, a rotary pump, a quartz glass tube, an infrared temperature measurement system (FTK9-R, Japan Sensor Corp.), the optical spectroscopy measurement system, two alumina combustion boats (located in the quartz glass tube), and the briquette and magnesium ribbon (placed in the alumina combustion boats)^32^. For the reaction described by Eq. (3), a microwave power of 1 kW was applied for 10 min to the TE102 waveguide cavity applicator under low vacuum (10 Pa) in the quartz glass tube. The optical measurements were carried out during the thermal processing.
